# Modifiable risk factors for perioperative hidden blood loss in unilateral biportal endoscopic surgery: a systematic review and meta-analysis

**DOI:** 10.20452/wiitm.2025.17986

**Published:** 2025-10-01

**Authors:** Zhiwu Zhang, Jiashen Shao, Hai Meng, Shuning Liu, Zihan Fan, Jisheng Lin, Qi Fei

**Affiliations:** Department of Orthopedics Beijing Friendship Hospital Capital Medical University Beijing China

**Keywords:** hidden blood loss, minimally‑invasive surgery, meta‑analysis, risk factors, unilateral biportal endoscopic surgery

## Abstract

**INTRODUCTION:**

Unilateral biportal endoscopic (UBE) surgery enables precise treatment of lumbar spine pathologies due to its inherent advantages typical of minimally‑invasive endoscopic procedures, including reduced intraoperative blood loss and minimal soft tissue dissection. However, hidden blood loss (HBL) remains a significant challenge in UBE, with limited data regarding its incidence and risk factors.

**AIM:**

This study aimed to investigate risk factors associated with HBL in UBE surgery.

**MATERIALS AND METHODS:**

Original studies evaluating risk factors for HBL in UBE surgery were systematically searched in MEDLINE, Embase, China National Knowledge Infrastructure, Wanfang Data, and the Cochrane Central Register of Controlled Trials (up to March 2025). The included studies met the quality assessment criteria of the Newcastle‑Ottawa Scale.

**RESULT:**

Six studies involving 601 patients subjected to lumbar UBE surgery were included. Our meta‑analysis identified that higher body mass index (BMI), prolonged surgical time, preoperative hypertension, and elevated preoperative hematocrit (HCT) levels were significant risk factors for increased HBL in UBE surgery (P <0.05). Sensitivity analysis confirmed the robustness of these findings, with no changes in the significance of the pooled results.

**CONCLUSION:**

Higher BMI, prolonged surgical time, preoperative hypertension, and elevated preoperative HCT levels are associated with an increased risk of HBL in patients undergoing lumbar UBE surgery. This study serves as a baseline reference for developing public health strategies to mitigate HBL in UBE procedures.

## INTRODUCTION 

Unilateral biportal endoscopic (UBE) surgery is an emerging minimally-invasive spinal surgery technique that enables precise treatment of lumbar spine pathologies by establishing 2 separate portals: 1 for endoscopic visualization and the other for surgical instrumentation.^1^;^2^;^3^;^4^ Initially pioneered by South Korean scholars,^5^ UBE technology has been progressively adopted in recent years across Asia, Europe, and North America, evolving into a standard procedure for managing degenerative spinal disorders, such as lumbar disc herniation and lumbar spinal stenosis.

Compared with conventional open surgery, UBE demonstrates the inherent advantages of minimally-invasive endoscopic spinal procedures, including reduced intraoperative blood loss and minimal soft tissue dissection.^6^;^7^ Hidden blood loss (HBL), a critical index for evaluating blood loss in endoscopic spinal surgeries, such as UBE, has gained increased recognition among spinal surgeons.^8^ HBL refers to a clinically significant postoperative decline in hemoglobin (Hb) and hematocrit (HCT) levels that cannot be fully accounted for by measurable intraoperative blood loss or postoperative drainage. This phenomenon typically results from unobserved blood loss attributable to intraoperative tissue oozing, postoperative blood sequestration in tissue spaces, and hemolysis.^9^ Although minimally-invasive techniques exhibit lower visible blood loss, HBL constitutes a substantial proportion of total blood loss and may predispose patients to postoperative complications, such as surgical site infections, anemia, and transfusion requirements,^10^;^11^ ultimately impacting clinical recovery and therapeutic outcomes. Currently, research on HBL in UBE procedures remains limited, predominantly comprising single-center, small-sample observational studies that have yielded inconsistent conclusions.

## AIM 

This study employed a meta-analytic approach to quantitatively synthesize existing evidence and systematically evaluate risk factors associated with HBL in UBE surgery. The findings are intended to provide high-level medical evidence for patient risk stratification and perioperative management protocols, which would ultimately guide the development of targeted strategies to reduce HBL-related complications.

## MATERIALS AND METHODS 

### Literature search 

A comprehensive computerized search was conducted across MEDLINE, Embase, China National Knowledge Infrastructure, Wanfang Data, and the Cochrane Central Register of Controlled Trials through March 2025 to identify studies investigating risk factors for HBL in UBE surgery. The search strategy incorporated the following key terms: (“unilateral biportal endoscopy” OR “UBE” OR “biportal endoscopic surgery” OR “biportal spine surgery” OR “biportal technique” OR “endoscopic spine surgery”) AND (“hidden blood loss” OR “occult blood loss” OR “unmeasured blood loss” OR “blood loss” OR “hemorrhage” OR “bleeding”) AND (“spine” OR “spinal” OR “lumbar” OR “decompression” OR “discectomy”). Manual screening of references from the retrieved articles was performed to identify additional eligible studies.

### Inclusion and exclusion criteria 

Two authors (ZZ and JS) individually assessed the titles, abstracts, and full texts of the searched publications to screen the appropriate articles for the meta-analysis. The inclusion criteria were as follows:

1. observational studies (cohort or case-control designs) or randomized controlled trials (RCTs);
2. studies exclusively involving patients who underwent UBE procedures for lumbar spine pathologies, specifically lumbar disc herniation and / or lumbar spinal stenosis. Studies combining UBE with other surgical techniques were excluded;
3. studies reporting HBL and its associated risk factors;
4. studies providing quantitative HBL data (eg, Hb or HCT level reduction) or significant analyses of relevant risk factors.

### Quality assessment

For observational studies, methodological quality was evaluated using the Newcastle-Ottawa Scale (NOS) which assesses 3 domains: selection of study groups (0–4 points), comparability between groups (0–2 points), and ascertainment of exposure / outcomes (0–3 points), with a maximum total score of 9. Studies scoring 6 points or more on the NOS were classified as high-quality. For RCTs, the Cochrane Risk of Bias Tool was employed to evaluate methodological rigor across 6 domains: random sequence generation, allocation concealment, blinding of participants / personnel, blinding of outcome assessment, incomplete outcome data, and selective reporting. Each domain was categorized as “low risk,” “high risk,” or “unclear risk.” The overall risk of bias was stratified as follows: low risk: all critical domains rated as low risk; moderate risk: at least 1 critical domain with unclear risk but no high-risk domains; high risk: at least 1 critical domain with high risk.

### Data extraction 

All data were independently extracted from eligible studies by 2 authors (ZZ and JS), using a standardized protocol. The following variables were collected from each study: first author’s name, publication year, country, HBL volume, identified risk factors, study design, and the number of cases. Discrepancies were resolved through discussion or third-party adjudication.

### Definition of hidden blood loss 

HBL was calculated using the following formula: HBL = total blood loss − visible blood loss.^12^;^13^ Total blood loss was estimated via the Gross or Nadler formula,^14^;^15^ based on preoperative-to-postoperative changes in Hb or HCT levels. Visible blood loss comprised intraoperative measurable blood loss (quantified using suction canisters and gauze weights) and postoperative drainage volume.

### Statistical analysis 

Adjusted odds ratios (ORs) with 95% CIs for each risk factor were extracted from original studies. Pooled analyses were performed to evaluate between-variable differences and identify risk factors for HBL. A *P* value below 0.05 was considered significant. Heterogeneity across the studies was assessed using the Q-test (*P* <⁠0.1) and quantified via the *I^2^* statistic, with thresholds interpreted as follows: *I^2^* below 50% indicating low heterogeneity, 50%–75% indicating moderate heterogeneity, and above 75% corresponding to high heterogeneity. A random-effects model was applied to calculate the pooled ORs when significant heterogeneity was detected (*P* <⁠0.1 or *I^2^* >50%). Otherwise, a fixed-effects model was used. Meta-analytic results were visualized using forest plots. Publication bias was not assessed due to the limited number of included studies (n <⁠10). Sensitivity analyses were conducted for variables with substantial heterogeneity by sequentially excluding outlier studies to investigate potential sources of heterogeneity. All analyses were performed using Stata, version 18 (StataCorp LLC., College Station, Texas, United States).

### Ethics 

This review article did not require approv- al of an ethics committee.

## RESULTS 

A total of 221 full-text records were retrieved, with 6 studies meeting eligibility criteriaFigure 1. Four studies were published in English and 2 in Chinese, spanning publication years from 2015 to 2025. These studies collectively analyzed 601 cases investigating risk factors for HBL in UBE surgery. Detailed characteristics of the included studies are presented in Table 1. NOS scores for observational studies were distributed as follows: 1 study scored 8 points,^16^ 3 scored 7,^17^;^18^;^19^ and 2 scored 6.^20^;^21^

**Figure 1:**
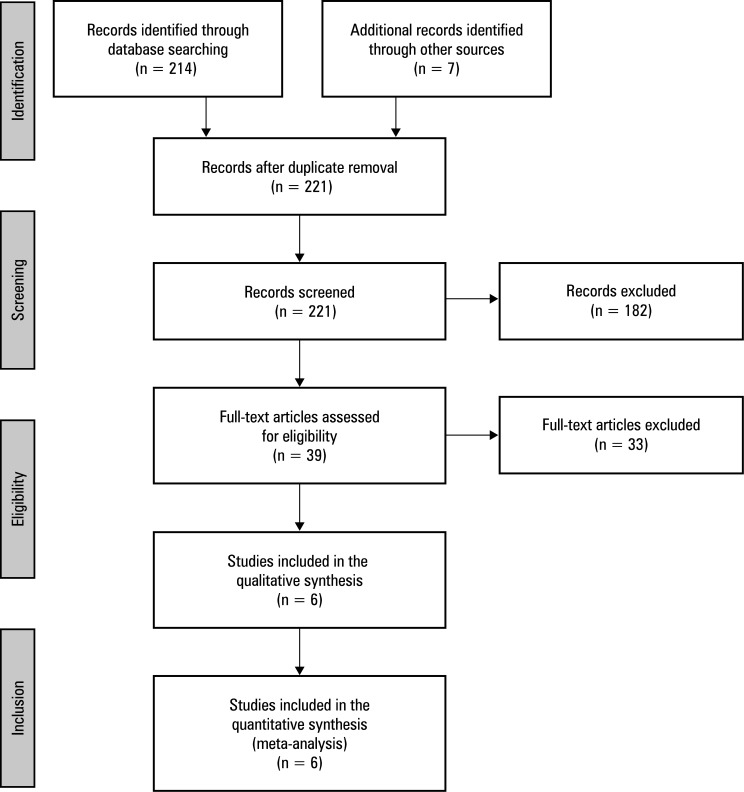
Literature selection process

**Figure 2:**
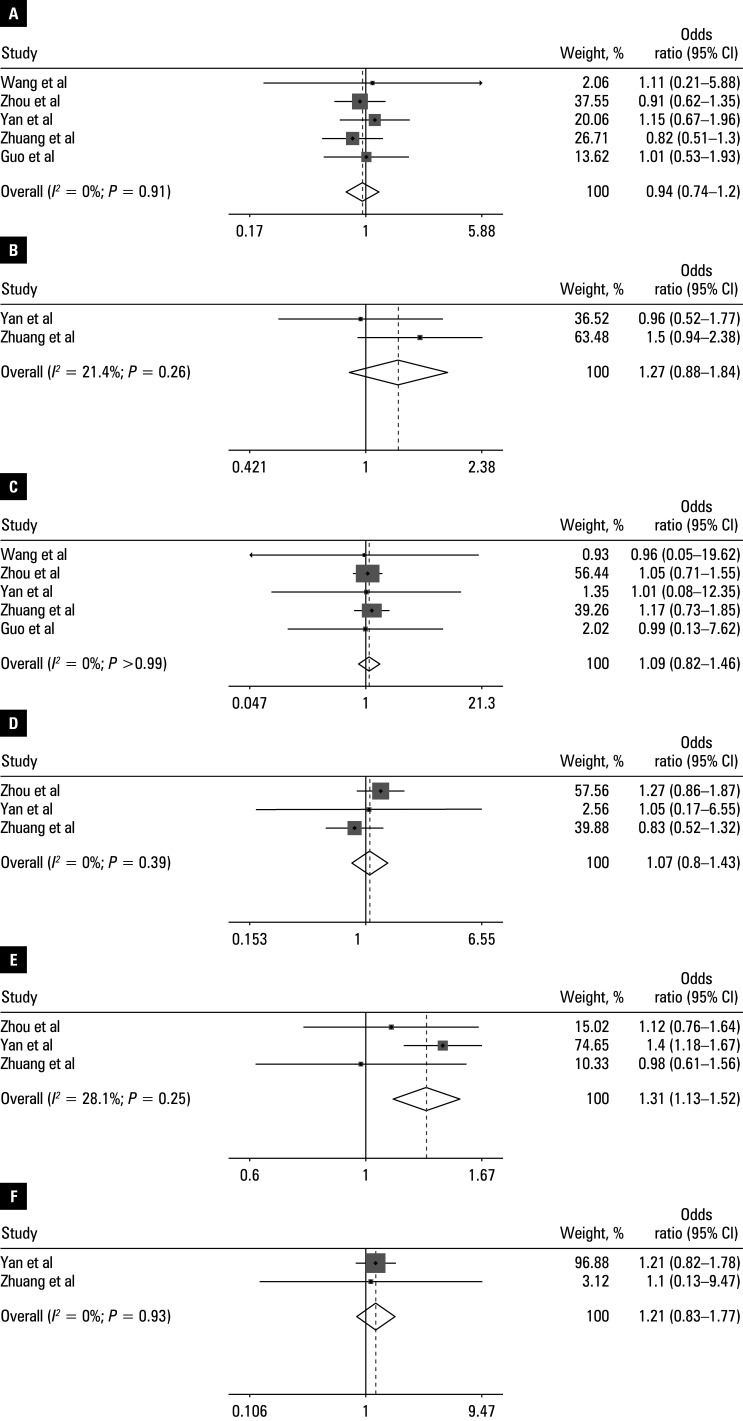
Forest plots of the meta-analysis of: **A** – age; **B** – preoperative ALB; **C** – preoperative APTT; **D** – ASA classification; **E** – BMI; **F** – diabetes. The horizontal line represents the 95% CI and each square represents the proportional weight of the study.

Abbreviations: see TABLE 1

**Figure 3:**
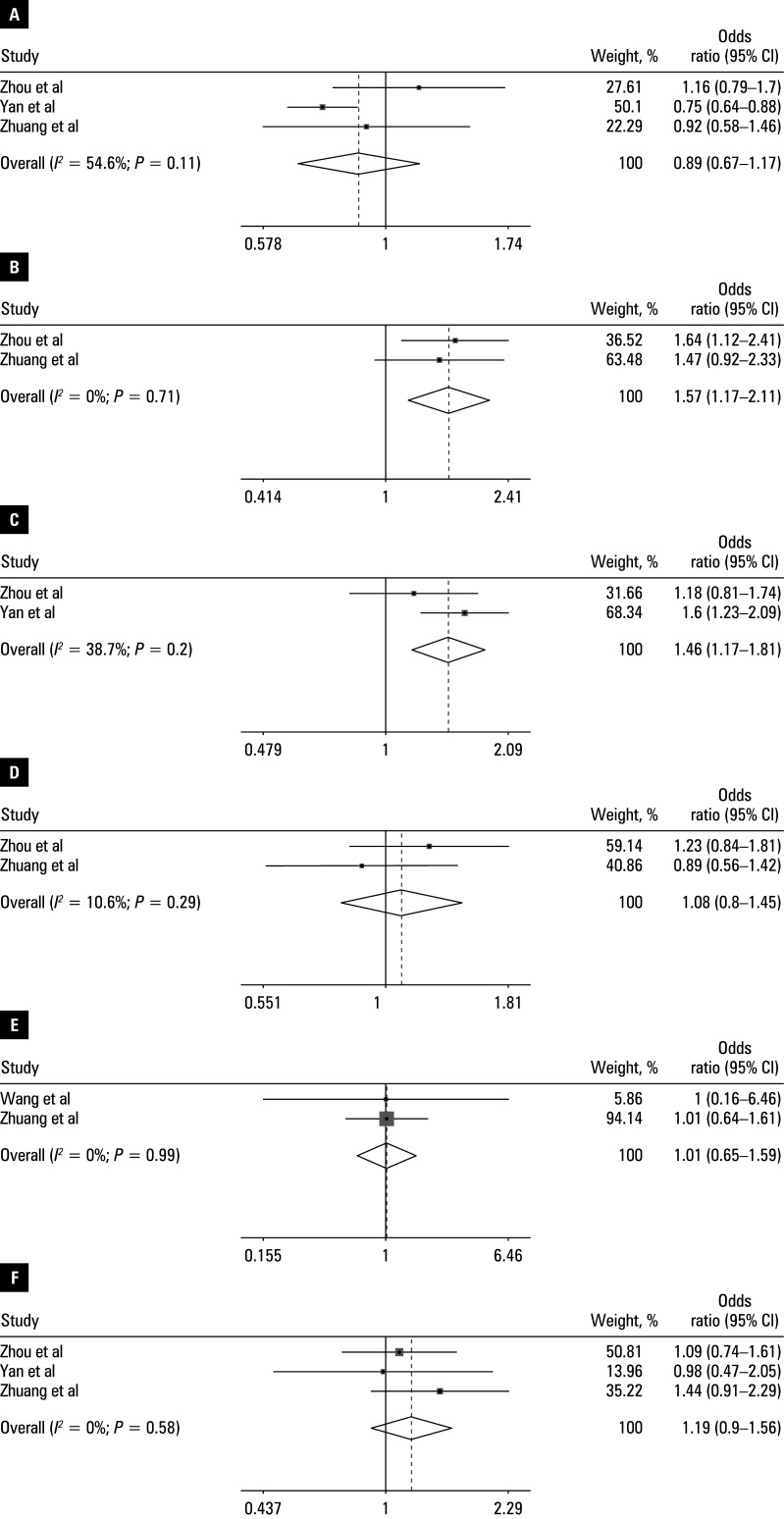
Forest plots of the meta-analysis of: **A** – preoperative FIB; **B** – preoperative HCT; **C** – hypertension; **D** – preoperative PLT; **E** – postoperative Hb; **F** – preoperative Hb. The horizontal line represents the 95% CI and each square represents the proportional weight of the study.

Abbreviations: see TABLE 1 and TABLE 2

**Figure 4:**
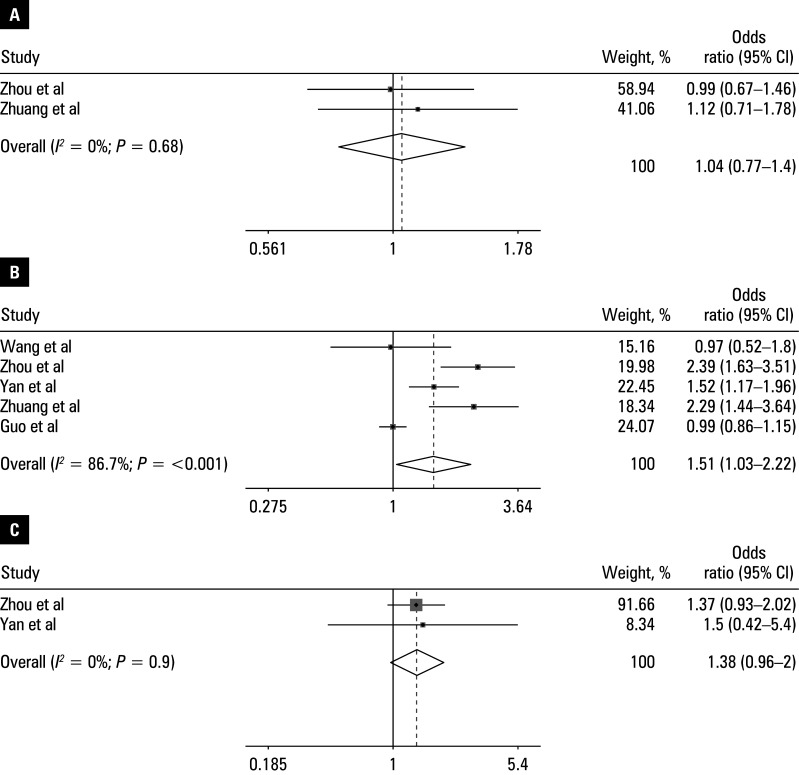
Forest plots of the meta-analysis of: **A** – preoperative PT; **B** – operative time; **C** – paraspinal muscle thickness. The horizontal line represents the 95% CI and each square represents the proportional weight of the study.

Abbreviations: see TABLE 1

Pooled OR ranged from 0.89 to 1.57 TABLE 2. Heterogeneity was observed for operative time (*I^2^* = 86.7%; *P* <⁠0.001). The following variables were identified as independent risk factors for UBE-associated HBL (all *P* <⁠0.05): body mass index (BMI; OR, 1.31; 95% CI, 1.13–1.52), operative time (OR, 1.51; 95% CI, 1.026–2.22); hypertension (OR, 1.46; 95% CI, 1.17–1.81), and preoperative HCT level (OR, 1.57; 95% CI, 1.11–2.11). No association was observed for: age (OR, 0.94; 95% CI, 0.74–1.2), preoperative albumin (OR, 1.27; 95% CI, 0.88–1.84), preoperative activated partial thromboplastin time (OR, 1.09; 95% CI, 0.82–1.46), American Society of Anesthesiologists classification (OR, 1.07; 95% CI, 0.8–1.43), diabetes (OR, 1.21; 95% CI, 0.83–1.77), preoperative fibrinogen level (OR, 0.89; 95% CI, 0.67–1.17), preoperative Hb level (OR, 1.19; 95% CI, 0.9–1.56), postoperative Hb level (OR, 1.01; 95% CI, 0.65–1.59), paraspinal muscle thickness (OR, 1.38; 95% CI, 0.96–2), preoperative platelet count (OR, 1.08; 95% CI, 0.8–1.45), and preoperative prothrombin time (OR, 1.04; 95% CI, 0.77–1.4). The main findings are presented in forest plots in Figure 2**,** Figure 3, Figure 4. 

Sensitivity analyses were performed on the risk factors with significant heterogeneity (operative time) by excluding low-quality studies or those with outlying effect estimates (ie, ORs and CIs that diverged substantially from the overall pooled estimate). Excluding these studies resulted in an *I^2^* below 50%, but the results of the meta-analysis did not change the significance, indicating that the pooled results were robust.

**TABLE 1:** Basic characteristics of the 6 included studies

First author	Publication year	Country	Patients, n	HBL, ml, mean (SD)	Study type	Identified risk factors for HBL
Wang ^19^	2021	China	136	469.5 (195.3)	Retrospective observational	Age, surgical segments, ASA classification, operative time, PBV, TBL, postoperative Hb, postoperative HCT, Hb decrease, HCT decrease, APTT, FIB
Yan ^20^	2023	China	200	295.5 (42.4)	Retrospective observational	ASA classification, hypertension, diabetes, sex, BMI, FIB, operative time, paraspinal muscle thickness
Zhou ^17^	2024	China	86	431 (160.52)	Retrospective observational	Operative time, preoperative HCT, ASA classification, paraspinal muscle thickness
Lin ^21^	2024	China	81	342.6 (285.6–412.4)**^a^**	Retrospective observational	Height, operative time, preoperative Hb, preoperative HCT, preoperative ALB, preoperative D-dimer, preoperative TC, preoperative TG, preoperative LDL-C, discectomy
Zhuang ^18^	2024	China	53	381.87 (218.01)	Retrospective observational	Operative time, preoperative Hb, preoperative HCT, preoperative ALB
Guo ^16^	2024	China	45	257.89 (190.66)	Retrospective observational	PT, soft tissue thickness, operative time, diabetes

**a **Data are presented as median (interquartile range).

Abbreviations: ALB, albumin; APTT, activated partial thromboplastin time; ASA, American Society of Anesthesiologists; BMI, body mass index; FIB, fibrinogen; Hb, hemoglobin; HCT, hematocrit; LDL-C, low-density lipoprotein cholesterol; PBV, predicted blood volume; PT, prothrombin time; TBL, total blood loss; TC, total cholesterol; TG, triglycerides

**TABLE 2:** Main outcomes of the meta‑analysis

Potential risk factors	No. of studies	Pooled OR	95% CI	*P* value	Q test, *P* value	*I2*c, %
Age	5	0.94	0.74–1.2	0.64a	0.91	
Preoperative ALB	2	1.27	0.88–1.84	0.2a	0.26	21.4
Preoperative APTT	5	1.09	0.82–1.46	0.56a	>0.99	
ASA classification	3	1.07	0.8–1.43	0.66a	0.39	
BMI	3	1.31	1.13–1.52	<0.001a	0.25	28.1
Diabetes	2	1.21	0.83–1.77	0.33a	0.93	
Preoperative FIB	3	0.89	0.67–1.17	0.39a	0.11	54.6
Preoperative Hb	3	1.19	0.9–1.56	0.22a	0.58	
Postoperative Hb	2	1.01	0.65–1.59	0.95a	0.99	
Hypertension	2	1.46	1.17–1.81	0.001a	0.2	38.7
Operation time	5	1.51	1.03–2.22	0.04b	<0.001	86.7
Paraspinal muscle thickness	2	1.38	0.96–2	0.09a	0.9	
Preoperative PLT	2	1.08	0.8–1.45	0.61a	0.29	10.6
Preoperative PT	2	1.04	0.77–1.4	0.79a	0.68	
Preoperative HCT	2	1.57	1.17–2.11	0.003a	0.71	

**a. **Fixed-effects model;

**b. **Random-effects model

**c**. *I^ 2^* statistic was defined as the proportion of heterogeneity not due to chance or random error.

Abbreviations: OR, odds ratio; PLT, platelet count; others, see TABLE 1

## DISCUSSION

The concept of HBL was first defined by ^22^ in 2000 as “unquantified blood loss occurring within the patient’s body, distinct from measurable intraoperative bleeding and postoperative drainage.” This meta-analysis aimed to identify risk factors for HBL in UBE lumbar surgery. The results demonstrated that BMI, hypertension, operative time, and preoperative HCT level were significant independent risk factors for HBL in UBE. In our study, BMI was identified as a significant risk factor for HBL, with low heterogeneity and robust results, which is consistent with numerous studies.^23^;^25^;^24^ used multivariate regression analysis to identify independent risk factors for HBL in intertrochanteric fractures, and developed a nomogram based on the results. Their findings indicated that BMI was an independent risk factor associated with HBL. A retrospective analysis ^23^ of 143 patients who underwent posterior lumbar fusion surgery reached the same conclusion. Patients with high BMI often have a thickened subcutaneous fat layer, making surgical field exposure difficult. This necessitates frequent adjustments of the endoscope angle and expansion of tissue dissection, thereby prolonging surgery time and increasing soft tissue injury. In addition, adipose tissue has a rich blood supply and is therefore more likely to bleed consistently when pulled or electrocauterized to stop bleeding. Notably, we found that surgical time itself was also an independent risk factor for increased HBL. The cancellous bone of the lumbar spine has a highly vascularized porous trabecular structure, where surface blood sinuses form a valveless low-pressure drainage system with the Batson venous plexus. Intraoperative curettage or burring exposes numerous open trabecular channels, leading to continuous bone surface bleeding as the surgical duration increases. Furthermore, the likelihood of leakage into interstitial spaces rises with prolonged surgery, allowing more blood to diffuse into the interstitial compartments and increasing ineffective blood loss.^26^;^27^ Additionally, prolonged surgery often involves more extensive tissue dissection and larger incisions, triggering the release of inflammatory mediators (such as interleukin-6 and tumor necrosis factor α) and tissue-type plasminogen activator. This activates the inflammation-fibrinolysis cascade, further exacerbating HBL. In our meta-analysis, surgical time was identified as an important risk factor associated with HBL, with significant heterogeneity (*I²* = 86.7%). The sources of significant heterogeneity are attributed to 2 main points. First, differences in surgeon experience considerably impact surgical time. Senior surgeons, with standardized workflows and greater proficiency in instrument handling, tend to perform surgeries faster than less experienced surgeons. Moreover, variations in the ability to manage complex anatomical structures, such as fat infiltration in patients with high BMI, further widen the range of surgical durations. Second, discrepancies in the definition of surgical duration across studies contribute to heterogeneity. Some studies measure surgical time from skin incision, while others start timing from endoscope insertion. Similarly, the end point of surgery varies, with some studies concluding at skin closure and others at the completion of dressing application. These inconsistencies in definitions reduce the comparability of surgical duration data, further increasing heterogeneity.

Several studies have shown that preoperative hypertension is another independent risk factor influencing HBL.^28^;^29^;^30^ This finding is consistent with our pooled results. In a hypertensive state, elevated blood pressure and increased capillary fragility cause persistent microvascular bleeding at the sites of soft tissue dissection and bone burring during surgery. This blood accumulates in the interstitial spaces, considerably increasing HBL. Additionally, cardiovascular regulation primarily depends on bioactive substances released by vascular endothelial cells, such as nitric oxide and prostacyclin. However, due to endothelial dysfunction in hypertensive patients, these vasoactive substances cannot be effectively secreted, leading to decreased vascular elasticity and vascular wall hardening.^31^ This pathological change prevents damaged blood vessels from properly retracting during surgery. As a result, even with postoperative drainage, blood that has seeped into the interstitial spaces is difficult to completely evacuate, further exacerbating HBL. Therefore, implementing controlled blood pressure reduction during the perioperative period is vital in limiting postoperative HBL in hypertensive patients. By optimizing blood pressure management, vascular function can be improved, reducing the risk of interstitial bleeding, and subsequently lowering the incidence of HBL.

Our study results indicate that preoperative HCT level is another independent risk factor associated with HBL. This finding is consistent with a study by Wang et al,^32^ who reported on risk factors for HBL in adolescent idiopathic scoliosis patients undergoing posterior spinal fusion surgery. Patients with higher preoperative HCT levels are often in a state of relative hemoconcentration. Increased blood viscosity leads to higher microcirculatory resistance, making red blood cells more likely to be retained and rupture at sites of vascular endothelial injury, such as bone burring surfaces. This process releases free Hb and heme, exacerbating oxidative stress and increasing vascular permeability, which may further contribute to HBL. Additionally, patients with higher HCT levels have a greater baseline red blood cell count. As a result, even with the same proportional blood loss, the decline in oxygen-carrying capacity is more pronounced, potentially leading to tissue hypoxia. This, in turn, may trigger microvascular dilation and leakage, further worsening HBL. The dilution effect of intraoperative intravenous fluid administration further lowers HCT levels, creating a cumulative effect on HBL and further elevating it. Based on these mechanisms, we hypothesized that higher preoperative HCT was closely associated with increased HBL. The underlying mechanisms likely involve intraoperative red blood cell loss, oxidative stress, changes in vascular permeability, and the dilution effect of fluid resuscitation. This finding underscores the importance of considering HCT levels during preoperative assessments to better predict surgery-related HBL risks and implement targeted interventions for optimized perioperative management.

This meta-analysis has some potential limitations. First, most of the included studies were retrospective and observational, which may impose selection and recall bias, and affect the statistical efficacy of the results. Second, there was some heterogeneity in the pooled results, influenced by factors, such as patient variability, standard of institutional care, and differences in surgical techniques of individual surgeons. Third, some studies did not provide ORs or adjusted ORs, and the pooled analyses performed by calculating the results may have affected the robustness of the conclusions. Finally, while our analysis identified several significant risk factors associated with occult blood loss in UBE surgery, we were unable to calculate specific cutoff values due to inherent limitations of the meta-analytic methodology. The original studies included in our analysis did not consistently report sufficient granular data to permit such calculations. Future primary studies with standardized measurements are needed to establish clinically meaningful thresholds for these risk factors.

## CONCLUSIONS 

In summary, this meta-analysis indicates that a higher BMI, prolonged surgical time, preoperative hypertension, and elevated preoperative HCT levels are significant risk factors associated with increased HBL in UBE surgery. Through identifying these risk factors and implementing targeted interventions, such as optimizing preoperative weight management, controlling blood pressure, dynamically monitoring HCT levels, and refining surgical procedures, clinicians can more effectively reduce the risk of HBL and prevent adverse outcomes associated with excessive blood loss. Furthermore, these findings provide a theoretical foundation for developing public health strategies, contributing to a more comprehensive perioperative management system that enhances the safety and efficacy of UBE surgery, and ultimately improves patient clinical outcomes.
